# Minutes and Proceedings of the Kentucky State Dental Association

**Published:** 1860-06

**Authors:** W. Muir Rogers, W. D. Stone

**Affiliations:** President


### Proceedings of Societies.

### MINUTES AND PROCEEDINGS OF THE KENTUCKY STATE DENTAL ASSOCIATION.

At a meeting of dentists of the State of Kentucky, convened at the Phœnix Hotel, in Lexington, on the 24th day of April, A. D. 1860, pursuant to a general call previously given, Dr. J. A. McClelland was called to the chair as President, *pro tem.*, and Dr. W. Muir Rogers appointed Secretary, *pro tem.*

On motion of Dr. W. D. Stone a committee was appointed to draft and report a form of Constitution for the Association. The Chair appointed Drs. A. S. Talbert, R. Peckover and S. F. Davis said committee. Whereupon said committee retired, and after consideration Dr. Talbert, Chairman, made a report which was received and the committee discharged. The Association then proceeded to take up the Constitution as reported which was adopted, article by article as follows, to wit:

Artice 1. This Society shall be called the "Kentucky State Dental Association."

Art. 2. The officers of this Association shall consist of a President, a Vice President, a Secretary, a Treasurer—also an Executive and an Examining Committee, each consisting of three members.

Art. 3. The officers shall be elected by ballot at each annual meeting, and their duties shall be such as appropriately belong to such offices in other deliberative bodies—a majority of votes only being necessary to a choice.

Art. 4. This Association now forming shall be chosen from members of this convention by ballot, two thirds of all the votes being necessary to an election.

Art. 5. After the forming of this Association all applicants for membership shall be recommended by the Examining Committee and be balloted for as mentioned in the preceding article.

ART. 6. Any member may be expelled from the Association for immoral conduct, mal-practice or other sufficient cause, on motion of an investigating committee appointed for that purpose, by a two thirds vote of the members present.

ART. 7. This Association shall be composed of two classes —Acting and Honorary members.

ART. 8. Each member at his election shall pay to the Treasurer of this Association the sum of two dollars, and the same amount annually thereafter; and any member who shall fail to pay his annual dues for two successive years shall forfeit his membership.

ART. 9. No member of this Association shall take a student for a shorter period than two years, unless he shall have received previous instruction to equal that term.

ART. 10. The meetings of this Association shall be held at such time and place as the majority of the members present at each meeting may determine.

ART. 11. The Association may receive contributions in money, books or other property, which may be either used or sold in aid of its purposes.

ART. 12. Seven acting members shall be necessary to the transaction of business at the opening of any meeting.

ART. 13. In the event that a quorum can not be obtained at any regular meeting of the Association, the same time and place shall be adopted for the following year.

ART. 14. The President shall have the power to call a meeting of the Association at any time he may think proper.

ART. 15. It shall be the duty of the President to appoint some member or members of the Association to read at the ensuing meeting an essay or essays on any subject pertaining to the profession.

ART. 16. Constitution may be amended by a vote of two thirds of the members present at any regular meeting.

*Names of Members.*—A. S. Talbert, Lexington, Kentucky; W. W. Justice, Winchester, Ky.; Stoddard Driggs, Lexington, Ky.; Edm. J. Peckover, Nicholasville, Ky.; W. Muir Rogers, Shelbyville, Ky.; W. N. Moffett, Lexington, Ky.; J. J. Wilson, Lexington Ky.; R. Peckover, Paris, Ky.; John Holmes, Georgetown, Ky.; W. D. Stone, Lexington, Ky.; James W. Grant, Lancaster, Ky.; J. A. McClelland, Louisville, Ky.

The Association then proceeded to the election of members.

On motion, adjourned till 8 o'clock P. M.

TUESDAY EVENING, 8 O'CLOCK.

The Association went into the election of officers, whereupon the following gentlemen were declared duly elected, viz:

Dr. W. D. Stone, *President.*
" Richard Peckover, *Vice President.*
" W. Muir Rogers, *Secretary.*
" J. A. McClelland, *Treasurer.*

*Executive Committee.*—J. W. Grant, E. J. Peckover, W. Muir Rogers.

*Examining Committee.*—Stoddard Driggs, A. S. Talbert, J. W. W. Justice.

The Chairman then appointed Dr. R. Peckover and John T. Toland to conduct the President, elect, to the chair.

The Committee appointed to draft a list of subjects for discussion made their report which was received and adopted.

SUBJECTS OF DISCUSSION.

I. Filling Teeth.
II. Fang Filling.
III. Treatment of Alveolar Abscess Plate Work.
IV. Vulcanite Base—Amber Base.
V. Partial Dentures for Lower Jaw.
VI. Articulation of the Jaws for full Dentures.
VII. Miscellaneous.

On motion, Dr. George F. Foote was elected an honorary member of this Association.

On motion of Dr. Justice the time for speaking is limited to ten minutes to each member until all have spoken.

By consent the regular order of discussion is deferred until to-morrow and the Association went into a general discussion. Then adjourned until 9 o'clock A. M., to-morrow.

WEDNESDAY, 9 O'CLOCK A. M.

The Association met pursuant to adjournment. The preceding minutes were read and approved and the subject of Filling Teeth discussed.

On motion, the regular discussion was suspended and Dr. John Holmes, of Georgetown, Ky., was recommended by the Examining Committee for membership and was duly elected.

Communications from Dr. Isaiah Forbes of St. Louis and Dr. W. H. Atkinson of Cleveland, expressive of their sym pathy and approval of the objects of this Society, were read and kindly received.

Dr. Richard Peckover of Paris, presented specimens of impressions of the mouth in a preparation of Paraffine, and a committee was appointed to investigate the fitness of this substance for dental purposes. Then adjourned until 2 o'clock P. M.

WEDNESDAY, 2 O'CLOCK P. M.

Met pursuant to adjournment. The committee appointed to consider the claims of Paraffine as a material for taking impressions and making patterns for Vulcanite Base, submitted their report which was received and placed on file.

*Report of Committee on Paraffine.*—The committee appointed to investigate the qualities of prepared Paraffine for taking impressions and making patterns for Vulcanite Base, submit the following report: It takes impressions sharp and from its exceedingly smooth property does not drag on being removed. The patterns for Vulcanite Base it would seem to answer better than Gutta Percha for the following reasons: It is easily adapted to the cast and does not spring from place as the Gutta Percha is apt to do.

J. A. McClelland,<br>A. S. Talbert,<br>W. W. Justice. } *Committee.*

A motion was made and unanimously carried, that the thanks of this Association be tendered Dr. Richard Peckover for the presentation of the Preparation of Paraffine to the consideration of this Association.

The subject of Fang Filling and of Alveolar Abscess were discussed, and Dr. Foote and J. T. Toland each made remarks with specimens and illustrations upon the process of making the hard Rubber Vulcanite and other Vulcanite Bases for artificial dentures. After which the remaining subjects were discussed in order.

On motion, *Resolved*, that when we adjourn we adjourn to meet in the city of Louisville, Ky., on the second Tuesday in April, 1861.

The Chair appointed Drs. J. A. McClelland, A. S. Talbert and W. Muir Rogers to read essays at the next meeting.

*Resolved*, That the Secretary is hereby instructed to prepare and furnish a copy of the minutes and proceedings of this Association to the *Dental Register* for publication.

*Resolved*, That the thanks of this Association be and are hereby tendered to the proprietors of the Phœnix Hotel for the gratuitous use of this room during our several sittings.

Drs. A. S. Talbert, J. A. McClelland and W. Muir Rogers are appointed Delegates to the National Convention to meet in Washington on the last Tuesday in July next, with authority to appoint substitutes.

The minutes were read and adopted.

On motion, the Association adjourned to meet in the city of Louisville, as before resolved.

